# Thermal Conversion Characteristics of Molasses

**DOI:** 10.1021/acsomega.1c03024

**Published:** 2021-08-10

**Authors:** Meheretu Jaleta Dirbeba, Anders Brink, Daniel Lindberg, Mikko Hupa, Leena Hupa

**Affiliations:** † Johan Gadolin Process Chemistry Centre, Åbo Akademi University, Henrikinkatu 2, Turku/Åbo 20500, Finland; ‡ Department of Chemical and Metallurgical Engineering, Aalto University, Kemistintie 1, P.O. Box 11000, Aalto, Espoo 00076, Finland

## Abstract

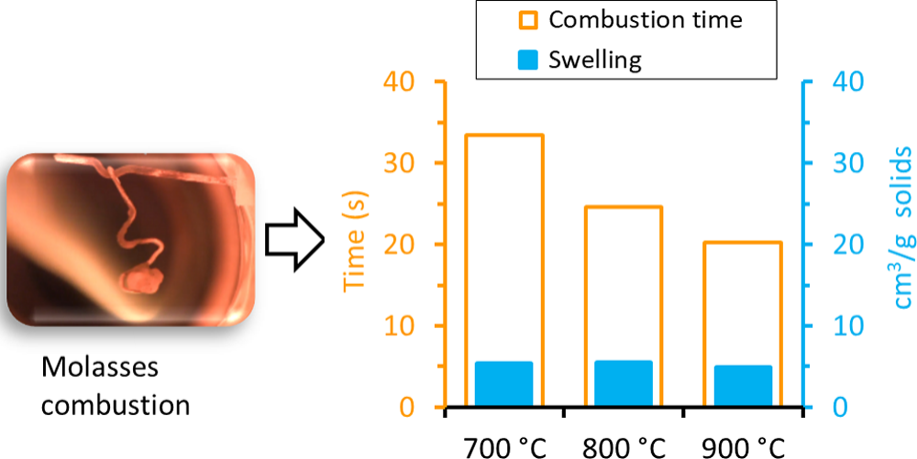

Agroindustrial biomass residues are considered potential feedstocks for renewable fuels and chemical production through processes such as thermal conversion. In this regard, thermal conversion characteristics of molasses, a byproduct from sugar production, have not been investigated. In this study, thermal conversion properties of molasses at temperatures of 700–900 °C have been studied using a single-particle reactor. Fuel swelling, combustion times, CO gas yields and gasification reactivities, and NO emissions and release of K and Cl during combustion and gasification were the thermal conversion characteristics of the molasses studied. In addition, the melting behavior of molasses ash produced at 500 °C was assessed using FactSage thermodynamic modeling and differential scanning calorimetry–thermogravimetric analysis measurements. Results of the molasses thermal conversion properties were compared with those of vinasse and black liquor samples from the integrated sugar–ethanol mill and soda pulping of hardwood, respectively. The results show that the molasses droplets had the least swelling tendency and the longest combustion time in the temperature range used, suggesting a lower conversion rate of molasses in an industrial boiler than the vinasse and black liquor. Moreover, at temperatures relevant for industrial gasification processes, that is, ≥800 °C, the gasification rates of molasses were lower than those of the vinasse and black liquor, probably owing to the lower total concentration of catalytic alkali and alkaline earth metals in the molasses. The release of K and Cl to a high degree from molasses during combustion and gasification and the low melting temperature of molasses ash make it a challenging fuel to utilize using the current thermal conversion technologies. Nevertheless, a black liquor recovery boiler type with a simpler (or an oxidizing) lower furnace than that of a black liquor recovery boiler and an entrained flow gasifier of the type demonstrated for black liquor may be potential options for the production of energy and recovery of inorganic chemicals from molasses.

## 1 Introduction

Agroindustrial biomass residues, forest residues, and waste streams containing biomass, including municipal solid waste, are increasingly gaining attention as sustainable feedstocks for the production of renewable fuels and chemicals. The utilization of these feedstocks for the production of energy and chemicals contributes to curbing CO_2_ emissions, reduces the burden on the environment and economy arising from their treatment and disposal, and promotes a circular economy. Consequently, the EU has issued a renewable energy directive, EU RED II,^1^ which promotes the use of these renewable sources for the production of fuels and chemicals.

**Figure 1:**
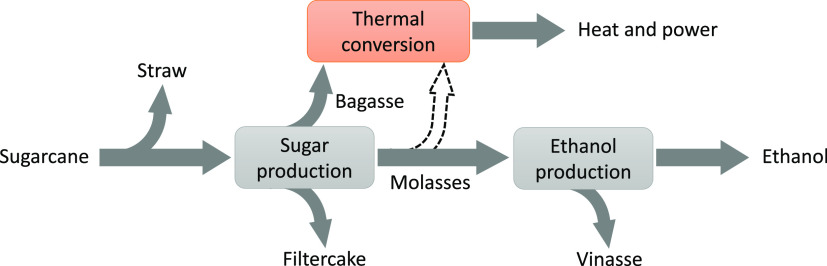
Simplified integrated sugar–ethanol process.

One of the agroindustrial processes where biomass residues have not been fully utilized is the integrated sugar and ethanol production.^2^Figure 1 shows a simplified version of the integrated process/mill. The dashed arrow in the figure indicates the proposed option in this study. Straw, bagasse, filtercake, molasses, and vinasse are the main biomass residues from the process. A detailed description of the process is available elsewhere.^2^ Here, only a brief description is presented.

The straw is left in the cane field unutilized or burned off from the cane stalks before harvesting. The cane stalks (stems) then undergo size reduction and extraction in the sugar mill to separate sucrose from the cane fibers or bagasse as juice. Most of the bagasse is combusted in a boiler to generate heat and power for the mill. The raw juice from the extraction unit is subjected to physical and chemical treatments to remove the impurities in it as a filtercake, a phosphorus-rich residue used mainly as a soil conditioner. Finally, the treated juice is concentrated by evaporation, and sucrose is recovered from the concentrated juice after crystallization and separation of the sugar crystals from the viscous mother liquor, molasses. The molasses is mainly used for ethanol production by first fermenting the residual sugars in it and then distilling ethanol from the fermented mass. The distillation step generates a dilute effluent called vinasse or stillage.

Molasses is characterized by a dark brown color, a slightly acidic pH (pH = 5–6), being viscous, having a typical solids content of 75–85 wt %, and containing a substantial ash fraction, typically 10–20 on a wt % dry molasses basis.^3^ The main organic components of molasses are sugars, oligosaccharides, polysaccharides, proteins, and organic acids, while potassium, calcium, chlorine, and sulfur are the dominant inorganic elements present in the molasses.^2,3^ In addition, molasses has a lower heating value of about 14 MJ/kg dry molasses,^2^ and it is produced at a rate of 3.5–4 tonnes of wet molasses per 100 tonnes of wet cane.^3^ Based on the total world sugarcane production of 1.91·10^3^ Mt (million tonnes) in 2019,^4^ the average annual molasses potential availability can be estimated to be approximately 70 Mt (wet basis). This would be equivalent to roughly 225 TWh of thermal energy supply per year. Maximum annual molasses throughput is obtained when all the sugarcane harvested is used for sugar production, as suggested in Figure 1. However, at present, in addition to the molasses, about 20% of the annual global sugarcane harvested is directly used to produce ethanol.

Although ethanol is the most commonly used biofuel in transport, the overall energy efficiency, and thus the sustainability, of using molasses as a feedstock for ethanol production is questionable. This is because according to several studies, for example, refs (5−7), the overall energy return on investment (EROI) for ethanol production from molasses or sugarcane juice is lower than the minimum recommended EROI value, >3.0,^8^ for the state-of-the-art energy production systems. In addition, the vinasse generated from ethanol distillery is mainly used in fertirrigation—fertilizing the soil and simultaneously providing the soil with water. However, fertirrigation poses potential environmental problems, including greenhouse gas emission^9^ and soil and water contamination,^10^ and it alone cannot consume the total amount of vinasse produced. Consequently, alternative options for molasses utilization are being investigated. For instance, Fuess and co-workers^5,11^ in their recent study have suggested an anaerobic biodigestion process for biohydrogen production from molasses as an alternative to the conventional ethanol process. Another option is thermal conversion, such as pyrolysis, gasification, or combustion, of the molasses. The advantage of these processes is not only converting the organic fraction of the molasses into heat and power, fuels, or chemicals but also avoiding vinasse generation. Also, the ash from these processes can be recovered and returned to the soil as a fertilizer.

Molasses shares some fuel properties with vinasse from the integrated sugar–ethanol process (Figure 1) and black liquor from the kraft process (wood pulping mill). These properties include having a similar heating value on a dry solids basis and containing a significant ash fraction. However, a unique advantage of molasses over the vinasse and black liquor for a thermal conversion process is that additional energy is not required to concentrate it to a high solids content. Thermal conversion characteristics of vinasse have been investigated,^12^ and the technologies for black liquor combustion and gasification are already well established (kraft recovery boiler) or demonstrated (entrained flow gasifier). However, to the best of the authors’ knowledge, thermal conversion of molasses has not been studied so far.

A critical property for the design, development, and operation of thermal conversion processes for fuels, like molasses, is their swelling tendency during thermal conversion. For black liquor, it had been shown that swelling plays a vital role in its conversion rate during combustion in a kraft recovery boiler and in influencing the trajectory of black liquor droplets in the boiler.^13−15^ Black liquor droplets with a high swelling tendency are lighter and burn faster while being carried over with the combustion gases, whereas those with a less swelling tendency are denser and slowly converted in the char bed.

Another factor important for the design and development of thermal conversion processes for biomass fuels is the type and concentration of ash-forming elements in the fuels. In particular, high concentrations of ash-forming elements, especially K and Cl, in biomasses are the main cause of ash-related problems in thermal conversion systems. These ash-related problems include ash deposit formation, bed agglomeration, corrosion, and slagging.^16^ In addition to the concentration of ash and ash-forming elements in biomass, the melting behavior of ash from the biomass determines the propensity of ash deposit formation and growth in boiler superheater tubes.^17^ The characteristic ash-melting temperatures are the initial or first melting (*T*_0_), sticky (*T*_15_), flow (*T*_70_), and complete melting (*T*_100_) temperatures.^17,18^ These characteristic temperatures determine the tendency of ash deposit formation and growth, corrosion, bed agglomeration, and slagging in thermal conversion processes.^17^ The melting properties of vinasse and black liquor ashes have been studied, and data on their characteristic ash-melting temperatures are available in the literature.^13,17−19^ However, literature information on the melting behavior of molasses ash is unavailable.

Still, a crucial parameter that needs to be considered in the design and development of thermal conversion technologies for biomasses is SO*_x_* and NO*_x_* emissions. Mechanisms for SO*_x_* and NO*_x_* formation during thermal conversion of biomass are well studied (see more details in the review study by Hupa et al.^20^). In addition, emission control measures for SO*_x_* and NO*_x_* are well developed. However, the NO*_x_* emission control technologies, such as selective noncatalytic reduction (SNCR) and selective catalytic reduction (SCR) systems,^21,22^ are more complicated and expensive than those for SO*_x_*. Thus, fuel-specific NO*_x_* emission data are required for adapting (or devising) a suitable NO*_x_* control technology.

The objective of this work is to shed more light on the properties of molasses for thermal conversion processes. Swelling tendencies, combustion times, CO gas yields and gasification rates, release of K and Cl, and NO emissions were the combustion and gasification characteristics of the molasses studied. In addition, the melting behavior of molasses ash was investigated. The aim is to provide the necessary information for modeling, designing, and developing industrial-scale thermal conversion processes for molasses with high conversion efficiency and low emissions. The combustion and gasification properties were determined by conducting combustion/gasification experiments in a lab-scale quartz glass reactor, also referred to as a single-particle reactor (SPR), and analyzing the composition of the ashes and gases from the experiments. The ash-melting behavior was assessed using FactSage thermodynamic calculations and differential scanning calorimetry (DSC)–thermogravimetric analysis (TGA) measurements. Results of the molasses thermal conversion characteristics were compared with those of vinasse and black liquor after conducting experiments using samples of the vinasse and black liquor under similar experimental conditions as with those of the molasses.

## 2 Experimental Section

### 2.1 Collection and Preparation of the Fuel Samples

Three agroindustrial biomass residues were used in this study: cane molasses, cane vinasse, and black liquor. The molasses and vinasse samples were obtained from a sugar mill in Ethiopia, while the black liquor, a byproduct in the soda pulping of hardwood, sample was obtained from a pulp mill in the USA. For practical reasons, the vinasse sample was dried at the mill at 105 °C for 24 h before shipping, whereas the molasses and black liquor samples were received concentrated to a dry solids content of about 75 wt %. The latter fuel samples were dried in the laboratory. All the dried samples were ground to <1 mm particle size. A further drying test performed in the laboratory for the dried and ground samples showed that the dried and ground molasses and vinasse samples had 13 and 7 wt % moisture contents, respectively, while the black liquor sample was completely dry. The drying test method was adapted from the TAPPI/ANSI Test Method T 650 om-15, which is used to determine the dry solids content of concentrated black liquor. Description of the method for the drying test is available in ref (23).

### 2.2 Proximate and Ultimate Analyses of the Fuels

The proximate analysis involves the determination of moisture (*M*), volatile matter (VM), fixed carbon (FC), and ash (ASH) contents of solid fossil or biomass fuels. The standard method, ASTM E870–82(2019), for the determination of VM contents of solid fuels requires heating samples of the fuels to 950 °C in a furnace for 7 min. However, heating the molasses, vinasse, or black liquor samples to such a high temperature would yield unreliable VM values. This is because the samples contain a substantial ash fraction, and as a result, the VM values obtained at 950 °C will be in part due to the release of significant levels of ash-forming matter from the samples. For instance, our previous study^19^ showed that about 5 wt % dry fuel basis mass loss during combustion of dried vinasse at 900 °C in the SPR was due to the release of ash-forming elements. For this reason, the proximate analysis of the fuel samples used in this work was carried out according to the method developed by Torquato et al.^24^ The method allows the determination of VM contents of solid fuels with high ash contents at a lower temperature, 600 °C, and in a CO_2_ gas atmosphere using a DSC–TGA setup.

The proximate analysis of the fuel samples used in this study was conducted even at a lower temperature, 500 °C, than that recommended by Torquato et al. A lower temperature was used to minimize loss of ash-forming matter from the fuels since it was observed in our previous study^25^ that significant levels of K and Cl were released at 600 °C from vinasse in CO_2_. However, at 500 °C, alkali release from the vinasse was not significant, even under N_2_ gas conditions.^26^ Moreover, the proximate analysis was conducted using the SPR instead of a DSC–TGA since the samples cannot be put in a DSC–TGA furnace due to safety reasons. The SPR is a quartz glass reactor enclosed in an electrically heated furnace. It is provided with a sample insertion probe and reaction gas supply ports. Detailed description of the SPR is available elsewhere.^27,28^

The *M* contents of the fuels were determined as follows. First, about 50–55 mg of the dried and ground fuel sample was weighed in a quartz glass sample holder and mounted onto the sample insertion probe. The sample was then inserted into the reactor, which was being purged with 99.99% pure CO_2_ gas (gas flow rate = 3.67 *l*_N_/min). Then, the temperature of the system was raised from room temperature to 105 °C, whereafter the system was kept isothermal at 105 °C until no further sample weight change was observed. Finally, the sample was withdrawn from the hot reactor, cooled in CO_2_ gas, and weighed. The decrease in the sample weight at 105 °C was due to loss of moisture from the sample, and the value was reported on a wt % dry fuel basis as the *M* content.

**Figure 2:**
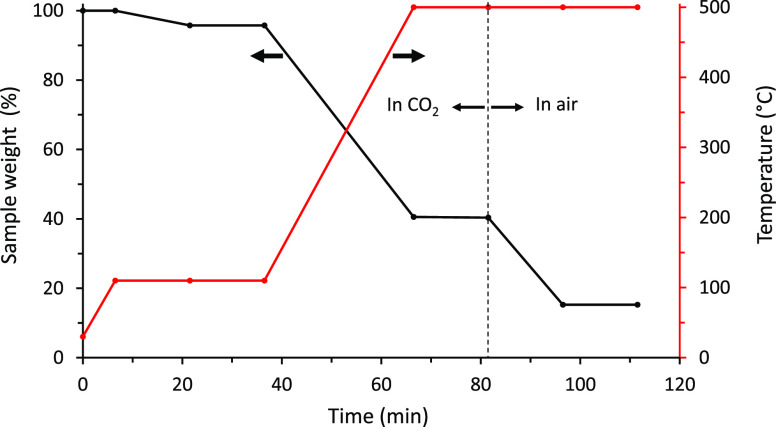
A typical run for the proximate analysis.

For the VM content of the sample, the temperature of the reactor with the sample from the drying stage in it was first increased from 105 to 500 °C. The system was then kept isothermal at 500 °C until the sample weight loss leveled off. The sample weight loss between 105 and 500 °C was reported on a wt % dry fuel basis as the VM content of the fuel. For determining the FC content of the fuel, the CO_2_ gas supply was shifted to air while maintaining the temperature of the system at 500 °C, and the system was run until the sample weight loss leveled off with only ash remaining. Here, the air was supplied to the system for burning off the carbon left in the sample. The weights of carbon burned off and the final residue (or ash) left in the system were reported on a wt % dry fuel basis as the FC and ASH contents of the fuel, respectively. Figure 2 shows a typical run for the proximate analysis.

The ultimate analysis of the fuels and determination of their heating values were carried out and reported previously,^2,23^ and the values are given in Table 2, Section 3.1.

### 2.3 Combustion and Gasification Experiments

The combustion and gasification characteristics of the fuels as a function of temperature were determined by combusting/gasifying samples of the fuels in the SPR, described in Section 2.2, at three different temperatures of 700, 800, and 900 °C. The gas atmosphere used in the SPR was 3 vol % O_2_/97 vol % N_2_ and 50 vol % CO_2_/50 vol % N_2_ for the combustion and gasification experiments, respectively. The total gas flow rate from the SPR was maintained at 3.67 *l*_N_/min under both gas conditions. For the combustion experiments, droplets of the fuel samples were used. The droplets were prepared by diluting the dried and ground fuel samples to 75–85 wt % dry solids content with ultrapure water. The combustion experiments were carried out as follows. The SPR was first heated to the desired temperature of 700, 800, or 900 °C. About 10 mg of the droplet was then mounted on a thin platinum hook, and the platinum hook with the droplet on it was mounted on the sample insertion probe described in Section 2.2. Next, the sample was inserted from the relatively colder environment of the SPR into the hot reactor and combusted until the CO_2_ and NO gas concentrations in the exhaust gas were close to 0.0 vol %. Finally, after combustion of the sample was complete, the sample insertion probe was withdrawn from the reactor, and the hook with the final residue or ash on it was weighed to 0.01 mg accuracy. The experiments were repeated five times at each temperature, and the average values were reported. Repeating the experiments five times also enabled to collect enough ash for elemental analysis.

The procedures for the gasification experiments in the SPR were similar to those for the combustion experiments except for the following. (a) Instead of the platinum hooks, quartz glass sample holders with a porous bottom (pore size = 1 μm) were used. In this case, it was not possible to use the platinum hooks because the molasses droplets did not stay on them under gasification conditions. (b) About 20 mg of the dried and ground fuel samples were used. (c) The gasification experiments were stopped when the CO and NO readings from the gas analyzers were close to 0.0 vol %. (d) The gasification experiments were repeated three times at each temperature, and the average values were reported.

Depending on the temperature and fuel type, the time required for complete combustion of the droplets in the SPR was in the range of 50 to 150 s. However, the time for the gasification reaction to complete was longer, between 150 and 350 s.

### 2.4 Swelling during Pyrolysis and Duration of the Combustion Stage

The maximum swollen volumes of the fuel droplets during pyrolysis as a function of temperature were determined from the combustion experiments described in Section 2.3. For the determination, the events during combustion of the fuel droplets in the SPR were first recorded using a digital video camera. Then, image recognition was used to identify the swollen droplets. The image recognition result was then imported into an IRIS software program, which processes the data into a total number of pixels, determines a circle with an equivalent area, and calculates the volume of the swollen droplet assuming a sphere. Finally, the value for the volume is converted from px^3^ to cm^3^ using a conversion factor calculated from an object of a known size. All the results were reported as specific swollen volume in cm^3^/g dry solids. The shortcoming of this method is that the assumed spherical shape may not represent the actual shape of the swollen droplet. Nevertheless, it is an important tool for comparing the relative swelling tendencies of the different fuels during pyrolysis.

The droplet combustion time, which is the time elapsed between ignition of the droplet and char burnout in the SPR, was obtained by processing the video recording from the combustion experiments.

### 2.5 CO Gas Yields and Gasification Reactivities

**Figure 3:**
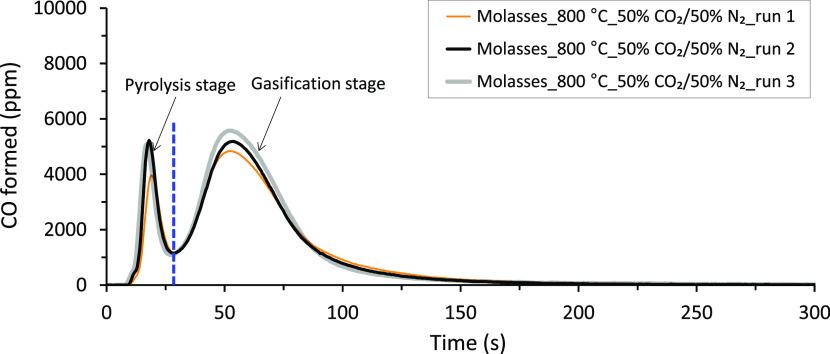
Typical runs showing CO gas formed as a function of time during the gasification experiments in the SPR. The vertical broken line indicates the time when the pyrolysis stage ended and the char conversion (gasification) stage started.

To determine CO gas yields as a function of temperature for the gasification experiments described in Section 2.3, the CO gas concentrations in the exhaust gas from the SPR were first measured using a nondispersive infrared CO gas analyzer from ABB (Germany). Prior to the gasification experiments, the analyzer was calibrated with known concentrations of CO gas. Figure 3 shows typical CO release profiles (in ppm) versus time (in s) obtained from the analyzer for the gasification experiments at 800 °C in the SPR. As seen from the figure, the repeatability of the experiments in the SPR was excellent. The two different CO gas emission peaks indicated in the figure correspond to the CO gas evolved during pyrolysis and gasification stages. The total CO gas yield from each gasification experiment was then obtained by integrating the area under the CO formed (ppm) versus time (s) and converting the value into a wt % dry fuel basis. For the conversion to a wt % dry fuel basis, the ideal gas law equation at atmospheric pressure and room temperature was used. In addition, the weight of the fuel droplet/sample used during the combustion/gasification experiments and the total gas flow rate from the SPR, 3.67 *l*_N_/min, were also used in the equation. Finally, the average results from the experiments repeated three times at each temperature were reported, on a wt % dry fuel basis, as CO gas yields.

The instantaneous gasification reactivities of the fuels at each temperature were determined based on the CO (ppm) formed during the gasification stage, as illustrated in Figure 3. For the determination of the reactivities, the minimum turning point of the CO emission profile, denoted by the vertical broken line shown in Figure 3, was first taken as the starting point for char conversion/gasification. Next, the total amount of CO formed (on a wt % dry fuel basis) during the gasification stage as a function of time was obtained in the same manner as described hereinabove for the CO gas yield. The total amount of char gasified (on a wt % dry fuel basis) as a function of time was then calculated based on the total amount of CO formed and using the Boudouard reaction given in eq 1. Finally, the instantaneous CO_2_ gasification rates were calculated using eq 2. In eq 2, *r*_i_ is the instantaneous gasification rate (in %/min), Δ*m* is the char weight change (on a wt % dry fuel basis) between measurement intervals of 0.5 s, *m_t_* is the char weight at time *t*, and Δ*t* is the time interval of 0.5 s.

**Figure 4:**
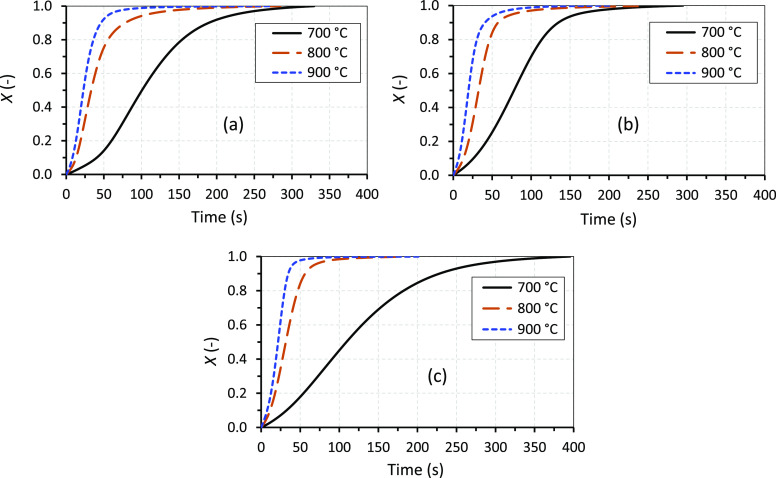
Char conversions with time at 700, 800, and 900 °C for molasses (a), vinasse (b), and black liquor (c).

The gasification reactivities of the fuels were compared after plotting the instantaneous gasification rates, obtained using eq 2, as a function char conversions, *X*. The char conversions as a function of time were calculated using eq 3. In eq 3, *m_t_* is the same as in eq 2 and *m*_0_ and *m*_f_ are the initial and final char weights on a wt % dry fuel basis, respectively. The final char weight, *m*_f_, is zero, whereas *m*_0_, the amount of char available at the onset of gasification, is equal to the amount of char consumed according to eq 1 after complete gasification of the char by CO_2_. Figure 4a–c shows the char conversions with time at the three different temperatures for the molasses, vinasse, and black liquor, respectively. According to Perander et al.,^29^ biomass char CO_2_ gasification reactions at temperatures ≤900 °C are limited by chemical kinetics. Thus, mass transfer limitations in the temperature range used in this study were not expected.
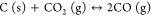
1
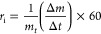
2
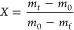
3

### 2.6 NO Emissions

**Figure 5:**
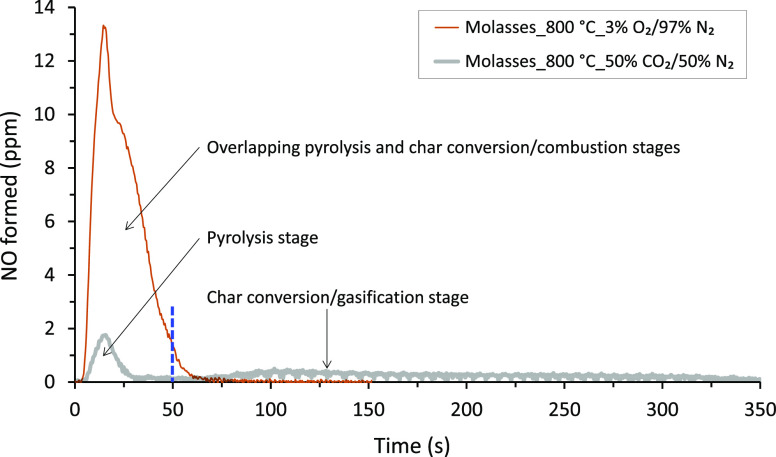
Typical runs showing NO gas formation as a function of time during the combustion and gasification experiments in the SPR. The vertical broken line indicates the time when the pyrolysis stage ended and the char conversion (gasification) stage started for the gasification experiment.

The NO gas emission levels from the fuels as a function of temperature during the combustion and gasification experiments described in Section 2.3 were determined by following similar procedures for the determination of the CO gas yields described in Section 2.5. However, in this case, the NO gas concentrations in the exhaust gas from the combustion/gasification experiments in the SPR were measured using a chemiluminescence NO gas analyzer from Teledyne (USA), and the analyzer was calibrated with known concentrations of NO gas before the experiments. Figure 5 shows typical NO emissions (ppm) as a function of time (s) obtained from the analyzer during the combustion and gasification experiments in the SPR. In addition to the NO gas yield, the NO release levels during the pyrolysis and char conversion stages for the gasification experiments were obtained by integrating the corresponding areas under the NO versus time emission curve indicated in the figure. For the combustion experiments, however, it was not possible to obtain the NO emission values for the devolatilization and char conversion stages separately since the two stages overlapped significantly. Overlapping of the two stages during the combustion experiments is seen in the figure as a shoulder in the NO versus time curve after the NO emission peak of the devolatilization stage. Such shoulders in the NO emission curves were observed only for the 700 and 800 °C combustion experiments, and they were broader for the 700 °C experiments than for the 800 °C.

### 2.7 Ash Yields and Elemental Analyses

The ash yields from the combustion and gasification experiments described in Section 2.3 were obtained from the weight difference between the fuel samples before combustion/gasification and their ashes after combustion/gasification. The average values from the combustion/gasification experiments repeated five/three times at each temperature were reported, on a wt % dry fuel basis, as ash yield results.

The ashes from the combustion and gasification experiments in the SPR were analyzed for their K, Na, and Cl contents. For the analyses, the ashes were first leached overnight separately in 250 mL of ultrapure water. The leachates (or solutions) were then analyzed for their alkali metal contents using inductively coupled plasma–optical emission spectroscopy (ICP–OES) (model Optima 5300 DV) from PerkinElmer (USA). The chloride contents of the leachates were analyzed using ion chromatography (IC) (model 881 Compact IC Pro) from Metrohm (Switzerland). The procedures for elemental analysis with the ICP–OES and Cl with the IC are available in ref (25). All the analyses were made in triplicates, and the average results on a wt % dry fuel basis were reported. The ash yield results listed in Table 4, Section 3.5, and amounts of the ashes (in mg) used for leaching were used to calculate the elemental analysis results from the ICP–OES and IC to a wt % dry fuel basis.

The molasses and black liquor ashes produced at 500 °C and used for assessing the ash-melting behavior, described in Section 2.9, were analyzed for their elemental compositions using scanning electron microscopy–energy-dispersive X-ray spectroscopy (SEM–EDX) (model 530 Gemini) from LEO (Germany). The procedures for the elemental analysis of the ashes with the SEM–EDX are available in ref (19), and the average elemental compositions of the ashes determined from the SEM–EDX analysis are given in Table 1, Section 2.9.

**Table 1:** Elemental Compositions (wt %) of the Molasses, Vinasse, and Black Liquor Ashes Produced at 500 °C

ash	elemental composition													
	C	H	N	O	K	Na	Ca	Mg	Si	Al	Fe	P	S	Cl
molasses	6.2	N.D.^a^	N.D.	36.2	27.3	0.8	8.6	1.2	1.4	0.2	N.D	0.2	6.5	11.6
vinasse^b^	7.2	N.D.	N.D.	38.8	27.2	1.1	9.1	1.4	1.6	0.2	0.2	0.3	4.8	14.1
black liquor	8.7	N.D.	N.D.	43.0	10.1	35.7	N.D.	N.D.	0.4	N.D.	N.D.	N.D.	0.3	1.8

a N.D.: not detected.

b Values are from ref (19).

### 2.8 Release of Alkali Metals and Chlorine

The levels of K, Na, and Cl released at each temperature from the fuels during the combustion and gasification experiments, described in Section 2.3, were determined from the difference between the concentrations of these elements in the fuels and their corresponding concentrations in the ashes. Elemental compositions of the fuels are given in Table 2, Section 3.1, whereas the K, Na, and Cl contents of the ashes from the combustion and gasification experiments are provided in Table 4, Section 3.5.

**Table 2:** Proximate Analysis Results and Elemental Composition and Heating Values of Molasses, Vinasse, and Black Liquor

original fuels	proximate (wt % fuel, dry basis)			ultimate (wt % fuel, dry basis)^a^														heating values (MJ/kg, dry fuel)^a^	
	VM	FC	ASH	C	H	N	O	K	Na	Ca	Mg	Si	Al	Fe	P	S	Cl	HHV	LHV
molasses	53	28	19	37.9	5.2	0.5	42.6	5.8	0.2	1.6	0.2	0.2	0.0	0.04	0.04	1.4	2.8	15	14
vinasse	52	15	33	32.9	4.5	1.0	36.4	14.1	0.4	3.2	0.6	0.6	0.03	0.1	0.1	2.7	6.2	14^b^	13^b^
black liquor	36	20	44	37.5	4.0	0.1	34.1	6.3	19.5	0.04	0.02	0.9	0.03	0.0	0.03	0.3	0.4	15	14

a Data for molasses, vinasse, and black liquor are from refs (2, 25, 23), respectively.

b Data are from ref (2).

### 2.9 Ash-Melting Behavior

The melting characteristics of molasses and black liquor ashes were assessed in the same manner as described in ref (19) for the vinasse. Here, only a brief description of the method is presented. The molasses and black liquor ashes were first produced at 500 °C in a muffle furnace according to the procedures given in ref (19). The ashing experiments were repeated three times for each fuel. Elemental compositions of the ashes were then analyzed using the SEM–EDX as per the procedures described in Section 2.7. The average elemental analysis results of the ashes from the ashing experiments repeated three times for each fuel are given in Table 1. For the vinasse ash, the elemental composition was taken from ref (19). The carbon contents of the ashes obtained from the SEM–EDX are most likely from carbonates present in the ashes. The presence of carbonates in the vinasse ash was verified using XRD analysis and reported previously.^19^ Finally, the characteristic ash-meting temperatures, that is, *T*_0_, *T*_15_, *T*_70_, and *T*_100_, for the ashes were calculated using FactSage version 7.2,^30^ and their *T*_0_ values were experimentally validated using a DSC–TGA (model SDT Q600) from TA Instruments (USA). Details of the experimental procedures for the measurement of *T*_0_ with the DSC–TGA are available in ref (19). For the calculations with FactSage, the elemental compositions of the ashes given in Table 1 and those of the fuels given in Table 2, Section 3.1, were used as inputs to the software. In addition, the following databases were used for the FactSage calculations: FTpulp for the alkali salt phases, FToxid for the molten slag phase and solid oxide/silicate phases, FactSage pure substance database for the gas components and other solid compounds, and the thermodynamic data published by Lindberg and Chartrand^31^ for the interaction of alkali salts with calcium and magnesium compounds in the molten salt phase.

## 3 Results and Discussion

### 3.1 Proximate and Ultimate Analyses

Table 2 lists the proximate analysis results and the elemental compositions (or ultimate analysis) and heating values of the fuels. The moisture contents of the dried and ground molasses and vinasse obtained from the proximate analysis were lower than those obtained from the drying test method described in Section 2.1. Since the latter method is more reliable than the former for determining the moisture contents of the fuel types used in this study (see Section 2.1), the values obtained from the latter method were used. Thus, the VM contents of the fuels given in the table were obtained after recalculating the VM results obtained from the proximate analysis to a wt % dry fuel basis by excluding the moisture contents.

As seen from the table, the molasses (also the vinasse) has a higher VM content than the black liquor on a wt % dry fuel basis. The high VM content of the molasses is most likely because it is mainly composed of low molecular weight organics, such as sugars,^3^ as described in the Introduction, chapter 1. However, the dominant organics in the black liquor are celluloses, hemicelluloses, and lignin,^13^ making it a less volatile fuel than the molasses during thermal conversion. For coal^32^ and other biomasses,^33^ the ignition temperature decreases with the increasing VM content. The high VM content of molasses suggests that it may ignite at a lower temperature than the black liquor during combustion. Moreover, Table 2 reveals that the molasses has a high FC content compared to the vinasse and black liquor, suggesting that it may yield more CO gas than the rest of the two fuels during gasification. Nevertheless, according to Basu,^34^ the VM content of a solid fuel, and consequently its FC content, varies considerably depending on the temperature at which it is determined and the heating rate used.

Furthermore, Table 2 shows that the molasses has similar C and H contents and heating values as those of the vinasse and black liquor. However, as seen from the table, the high levels of K and Cl in the molasses pose a potential challenge for utilizing it in conventional thermal conversion systems.

### 3.2 Swelling during Pyrolysis and Duration of the Combustion Stage

**Figure 6:**
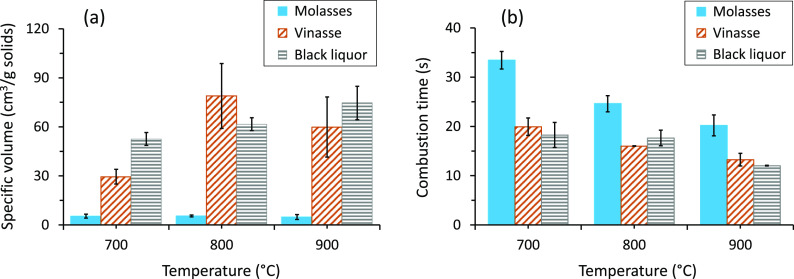
Results of specific swollen volume (a) and combustion time (b) as a function of temperature for molasses, vinasse, and black liquor droplets in 3 vol % O_2_/97 vol % N_2_ gas atmosphere in the SPR. Error bars are standard deviations calculated based on results from the experiments repeated five times at each temperature.

Figure 6a,b shows the results of the maximum specific swollen volume and combustion time, respectively, as a function of reactor temperature for combustion of the molasses, vinasse, and black liquor droplets in the SPR. The swelling results are for the pyrolysis stage of the combustion experiments in the SPR. The high error bars for the results shown in Figure 6a are related to the method used, especially the spherical representation of an irregular object.

As seen from Figure 6a, the maximum specific swollen volume of the fuels trended differently with temperature. The molasses had the least swollen volume compared to the vinasse and black liquor, and its swelling was independent of temperature in the temperature range used. For the vinasse, however, the swollen volume first increased by about a factor of two and a half when the temperature was raised from 700 to 800 °C, but it then decreased when the temperature was increased from 800 to 900 °C. The specific swollen volume for the black liquor slightly increased with temperature in the temperature range considered.

The low swelling tendency of the molasses was not expected given its higher VM content, which is similar to that of the vinasse (see Section 3.1). However, several studies, for example, refs (35−37), reported that swelling properties for similar fuels, such as black liquor, are influenced by various factors, including differences in viscosity and chemical composition. However, the influence of the different physical and chemical properties of molasses on its swelling tendency deserves a separate and dedicated study. Nonetheless, the low swelling tendency of the molasses has an important practical implication: thermal conversion of the molasses in an industrial boiler may be slower than those of the vinasse and black liquor (see Section 1). The slower conversion of the molasses was observed from the longer combustion time of its droplets in the SPR. Figure 6b shows combustion times for the molasses, vinasse, and black liquor droplets at the three different temperatures. As seen from the figure, the molasses droplets had a longer combustion time in the SPR at all temperatures compared to the vinasse and black liquor, owing to the low swelling tendency of the molasses droplets. An interesting aspect of the slow conversion of molasses is a lower chance of entrainment of ash particles with the flue gas during combustion of the molasses in a boiler. This is, however, advantageous from the perspective of minimizing ash-related problems in boiler superheater tubes, and at the same time, increasing the recovery of inorganic chemicals from the molasses with the bottom ash.

The results shown in Figure 6a,b further show the relative influence of swelling and temperature on the combustion time of the fuel droplets. At a given temperature, the fuel with the maximum swelling tendency had the shortest combustion time and vice versa. This trend is a well-established phenomenon for black liquor droplets.^13−15^ It is also well established for combustion of black liquor droplets that increasing the temperature in the temperature range relevant to recovery boilers decreases the combustion times of the droplets.^13−15^ However, as seen from the figures, the combustion time for all the fuels decreases with temperature regardless of the maximum specific swollen volume, indicating that temperature has an overriding effect on combustion time compared to swelling. However, more work is needed to validate this claim. The stronger influence of temperature on combustion time of the fuel droplets than swelling may be due to the fragmentation of the droplets at high temperatures before reaching maximum swelling, consequently leading to shorter combustion times.

### 3.3 CO Gas Yields and Gasification Reactivities

Table 3 shows the CO gas yield results on a wt % dry fuel basis for the molasses, vinasse, and black liquor gasification experiments in the SPR at 700, 800, and 900 °C described in Section 2.3. The results given in the table were obtained based on the procedures described in Section 2.5 and include the CO gas formed during the pyrolysis stage (see Figure 3, Section 2.5). The CO gas produced during the pyrolysis stage accounts for approximately 10 to 15% of the total CO gas yield at a given temperature. According to Ahmed and Gupta,^38^ the CO gas yield during biomass pyrolysis is almost independent of temperature. This indicates that the changes in the CO gas yields with temperature, given in Table 3, are mainly due to char gasification by CO_2_.

**Table 3:** CO Gas Yield Results (wt % Dry Fuel) for Molasses, Vinasse, and Black Liquor Gasification Experiments in the SPR at 700, 800, and 900 °C

	temperature (°C)		
sample	700	800	900
molasses	99	110	115
vinasse	71	75	89
black liquor	99	101	102

As seen from the table, the molasses had the highest CO gas yield, except at 700 °C, followed by the black liquor, whereas the vinasse had the lowest. At 700 °C, the molasses and black liquor yielded the same level of CO gas. The differences in the CO gas yields of the fuels under similar gasification conditions can be attributed to differences in their chemical compositions given in Table 2, Section 3.1. In particular, the high FC and low ash contents of the molasses are probably the cause for its high CO gas yield compared to those of the black liquor and vinasse. Moreover, in addition to the concentration of organics in biomass, several studies have reported that the syngas yield from biomass gasification varies depending on the type of organic constituents of the biomass. For example, Hanaoka et al.^39^ and Nam et al.^40^ have reported that gasification of a biomass fuel with a high lignin content yields less CO gas than gasification of cellulose-rich biomass. Similarly, the lower CO gas yield of the black liquor than that of the molasses may be due to the higher lignin content of the black liquor.

Table 3 further shows that the CO gas yield increased with temperature for all the fuels. The increase in the CO gas yield with temperature is higher for the molasses and vinasse than for the black liquor. The CO gas yield trends with temperature are similar to those reported in the literature for other biomasses.^38,41,42^ The increase in CO gas formation with temperature is mainly due to the increased char gasification reaction kinetics at higher temperatures, that is, the forward Boudouard reaction given in eq 1, Section 2.5, is favored at ≥800 °C. The CO gas formed at such high temperatures does not have sufficient residence time in the bulk of the char (or in the gas-phase product above the char surface) for oxidation to CO_2_ gas, and therefore, more CO gas is produced. At lower temperatures, however, the slow CO_2_ gasification reaction kinetics gives the CO formed ample time in the char (or in the gas-phase product) to be partially oxidized to CO_2_ gas. As a result, less CO gas yields were obtained at 700 °C than at 800 and 900 °C, as shown in Table 3. It is well known that metal oxides, such as Fe_2_O_3_,^43^ catalyze, and at the same time, serve as oxidants for CO oxidation to CO_2_ gas. Iron (Fe) was present in the molasses and vinasse samples used in this study (see Table 2, Section 3.1).

**Figure 7:**
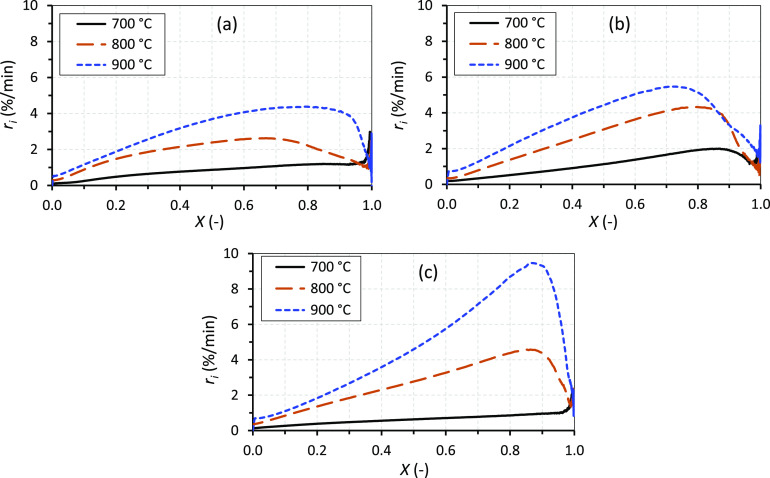
Instantaneous gasification rates as a function of conversion at 700, 800, and 900 °C for molasses (a), vinasse (b), and black liquor (c).

Figure 7a–c shows the instantaneous gasification rates, *r*_i_, as a function of conversion, *X*, at the three different temperatures for the molasses, vinasse, and black liquor chars, respectively. The results shown in the figures are average values from the experiments repeated three times at each temperature.

As seen from the figures, the gasification reactivity increased with temperature for all the chars, similar to the reactivity trends with temperature reported in the literature for other biomass chars.^44,45^ The increase in the gasification rates with temperature is related to the catalytic activity of inorganics in the chars and the morphological structures of the chars. Alkali and alkaline earth metals, especially K and Ca (the main cationic inorganic elements in biomass), are known to catalyze char gasification reactions, and their catalytic effect is more significant at higher temperatures. In addition, several studies, for example, refs (46, 47), have reported that devolatilization of biomass at high temperatures produces chars with macropores promoting gasification reactivity.

Figure 7a–c also shows that the gasification rates at 700 °C varied almost linearly throughout the char conversion for all the chars except for the vinasse char. For the vinasse char, the rate at 700 °C started to decrease after about 85% char conversion. For all the chars, the rate at 800 and 900 °C first increased linearly with char conversion until at least 60% conversion, then reached a plateau in the range of 70–90% char conversion, and finally decreased with char conversion. The increase in the reactivities of the chars with conversion is probably due to the increase in the concentrations of catalytic alkali and alkaline earth metal species in the chars as the chars were consumed by CO_2_. A similar observation was reported in our previous study^48^ for CO_2_ gasification reactivities of chars from agroindustrial biomass residues, indicating that a similar mechanism might have happened for the results shown in Figure 7a–c. While the attainment of a plateau in the rate curves given in the figures may be due to saturation of the chars with catalysts,^48^ the decrease in the reactivities at high char conversions may be explained by various mechanisms. These mechanisms include a decrease in the char surface area^49^ and deposition of impurities or products in the char pore structure.^29^

Figure 7a–c further shows that the molasses char had a lower reactivity, except at 700 °C, than the vinasse and black liquor chars, especially above char conversions of 40%. This agrees with the slow conversion of the molasses droplets during combustion (Section 3.2). The low char reactivity of the molasses is probably due to the low total concentration of catalytic alkali and alkaline earth metals in the fuel (Table 2, Section 3.1). On the contrary, the high Na + K concentration in the black liquor may be the cause for the high reactivity of the black liquor chars at 900 °C seen in Figure 7c.

### 3.4 NO Emissions

**Figure 8:**
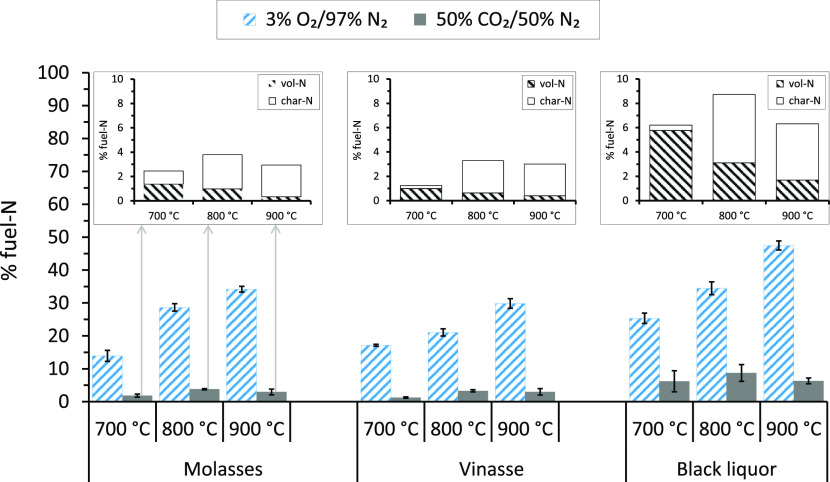
Results of fuel-N release as NO during combustion and gasification of molasses, vinasse, and black liquor at 700, 800, and 900 °C in the SPR. Error bars are standard deviations calculated based on results from the experiments repeated five or three times at each temperature.

Figure 8 shows the results of fuel-N release as NO during combustion and gasification of the molasses, vinasse, and black liquor samples in the SPR at 700, 800, and 900 °C. The results are given as percentages of fuel-N. Under both combustion and gasification conditions, the contribution of thermal NO to the NO emission results given in the figure can be considered negligible since the temperatures were low. In other words, thermal NO emissions become significant only at temperatures above 1400 °C. Thus, the NO emission results given in the figure originated mainly from the fuel-N. The inset figures shown in Figure 8 show the magnified version for the NO emissions during gasification of the fuels in the SPR. In the inset figures, the shares of fuel-N released as NO during the devolatilization and char conversion (gasification) stages are given as vol-N and char-N, respectively.

As seen from Figure 8, the fuel-N released as NO increased almost linearly with temperature for all the fuels under combustion conditions. For the molasses, about 15% of the fuel-N was released as NO at 700 °C, and the release level doubled when the temperature was increased from 700 to 800 °C and reached 35% of the fuel-N at 900 °C. The corresponding fuel-N percentages released were slightly lower for the vinasse, except at 700 °C, and higher for the black liquor compared to those for the molasses. The increase in the release of fuel-N as NO with temperature in the O_2_ gas atmosphere is attributed to the increased oxidation of the NO precursors, that is, NH_3_, HCN, and HNCO, with increased temperature. Previous studies^50,51^ have also reported increased fuel-N conversion to NO with temperature under combustion conditions. The extents of fuel-N released as NO from the molasses, vinasse, and black liquor samples might have been even higher than those given in the figure. This is because the concentration of the oxidant, 3 vol % O_2_, in the gas supplied to the SPR during the combustion experiments was not sufficient to oxidize the NO precursors fully. During the combustion experiments in the SPR, the CO gas analyzer showed the presence of CO in the exhaust gas from the reactor, indicating a substoichiometric gas condition in the reactor. At substoichiometric air conditions, the NO formed significantly decreases the release of fuel-N as NO.^51^

Moreover, Figure 8 shows that the CO_2_ gas atmosphere strongly suppressed the NO release: the fuel-N release as NO was below 5% of the fuel-N for the molasses and vinasse and less than 10% of the fuel-N for the black liquor at all temperatures. There are two likely causes for the low NO release levels during gasification of the fuels in the SPR. First, the relatively reducing CO_2_ gas conditions in the SPR than O_2_ might have decreased the oxidization of the NO precursors to NO in a similar manner described above for the substoichiometric gas conditions. Second, according to Vähä-Savo,^52^ CO_2_ gasification of black liquor and other fuels (peat, bark, and coal) impregnated with alkali carbonates and sulfates decreases the release of fuel-N as NO considerably due to the formation of alkali cyanate. A similar mechanism might have occurred for the gasification results shown in Figure 8.

A closer look at the results in CO_2_ shown in the inset figures in Figure 8 shows the differences in the fuel-N release trends with temperature during the devolatilization and char conversion stages. The volatile fuel-N, vol-N, release as NO during the pyrolysis stage linearly decreased with temperature for all the fuels. However, the char-N release as NO during the char conversion stage initially increased with temperature and then decreased when the temperature was increased from 800 to 900 °C. Such trends in the release of vol-N and char-N with temperature may be related to the increased formation and decomposition of cyanates during the devolatilization and char conversion stages, respectively. A similar observation was reported by Vähä-Savo.^52^ However, the lower char-N release as NO at the 900 °C experiments than at 800 °C may have resulted from increased secondary NO reactions in the gas phase at 900 °C.

Figure 8 further shows the influence of N concentration in the fuel samples on the fuel-N release as NO during combustion and gasification of the samples in the SPR. On a wt % dry fuel basis, the amount of fuel-N released as NO increased with the N contents of the fuels at all temperatures and under both gas conditions (see Figure S1 in the Supporting Information). The results agree with previous studies on black liquor.^52,53^ However, as can be observed from Figure 8, the percentage of fuel-N released as NO generally decreased with the N contents of the fuels. The highest percentage of fuel-N released was from the black liquor sample (with the least N content), while the least percentage released was from the vinasse (with the highest N content).

In general, the significant NO emissions under combustion conditions from the molasses indicate that industrial-scale combustion processes for molasses require incorporating suitable NO*_x_* control measures into the processes. Among the two main available primary NO*_x_* control methods, that is, air and fuel stagings, the air staging combustion for the molasses does not seem to be applicable. This is because air staging requires a boiler with a reducing gas atmosphere in the lower furnace, which increases alkali release (Section 3.6) from the molasses and consequently the risk of corrosion and ash deposition in the superheater tubes. On the contrary, fuel staging combustion where the fuel is fed into the boiler at two different locations (or levels) along the height of the boiler may be an option for the molasses. In this option, the molasses can be fed to the boiler at the first level, also known as the primary zone, and combusted with excess primary air. At the same time, the oxidizing gas condition in the primary zone retains most of the alkalis from the molasses in the bottom ash, as described in Section 3.6. The NO formed during combustion of the molasses can then be reduced to N_2_ by supplying an alkali-lean secondary fuel, such as oil or natural gas, to the combustion gases at the second level, also termed as the reburn zone. Finally, complete fuel burnout can be achieved by supplying additional air to the boiler in the final zone, farther above the secondary fuel feeding level. According to Salzmann and Nussbaumer,^54^ this process can reduce up to about 80% of the NO*_x_* formed during biomass combustion. In addition to fuel staging, secondary NO*_x_* control systems, including SCR and SNCR methods, can also be used to reduce NO*_x_* emissions from molasses combustion.

### 3.5 Ash Yield and Elemental Analysis Results

Table 4 lists the ash yield and Na, K, and Cl analysis results as a function of temperature for the molasses, vinasse, and black liquor ashes from the combustion and gasification experiments in the SPR. The results are given on a wt % dry fuel basis. The ash yield results given in the table and the amounts of the ashes obtained after combustion/gasification of the fuel samples in the SPR were used to convert the elemental analysis results obtained from the ICP–OES/IC to a wt % dry fuel basis. The Na analysis results for the molasses and vinasse ashes and Cl results for the black liquor ash were not reported since the results were not reliable. The unreliability of the results was mainly due to the low concentrations of these elements in the corresponding parent fuels, as given in Table 2, Section 3.1.

**Table 4:** Ash Yields and Na, K, and Cl Contents of the Ashes (wt % Fuel, Dry Basis)

		3% O_2_/97% N_2_			50% CO_2_/50% N_2_		
		700 °C	800 °C	900 °C	700 °C	800 °C	900 °C
molasses ash	ash yield	12.9	10.1	8.8	8.6	8.3	4.8
	K	4.7	2.9	2.1	3.2	0.5	0.5
	Cl	1.0	0.1	<0.05	1.6	0.1	<0.05
vinasse ash	ash yield	30.1	22.3	16.6	25.7	17.4	12.2
	K	11.5	8.0	4.5	9.4	4.2	1.5
	Cl	3.3	1.1	<0.05	4.6	1.4	<0.05
black liquor ash	ash yield	42.3	36.1	24.5	27.0	23.2	26.3
	K	4.6	3.5	2.6	3.2	1.3	0.7
	Na	15.4	12.3	9.1	13.5	6.2	5.2

As seen from the table, the ash yields and Na, K, and Cl contents of the ashes decreased when the temperature was increased from 700 to 900 °C under both combustion and gasification conditions. However, a higher ash yield result was obtained from the black liquor gasification at 900 °C than at 800 °C. The higher ash yield for the black liquor at 900 °C may be due to the formation of alkali carbonates from the organically bonded alkali metals of the black liquor. The formation of alkali carbonates at temperatures above 800 °C during CO_2_ gasification of spruce wood chars^29^ and pyrolysis of lignin^55^ in a CO_2_ gas atmosphere have been reported.

### 3.6 Release of Alkali Metals and Chloride

**Figure 9:**
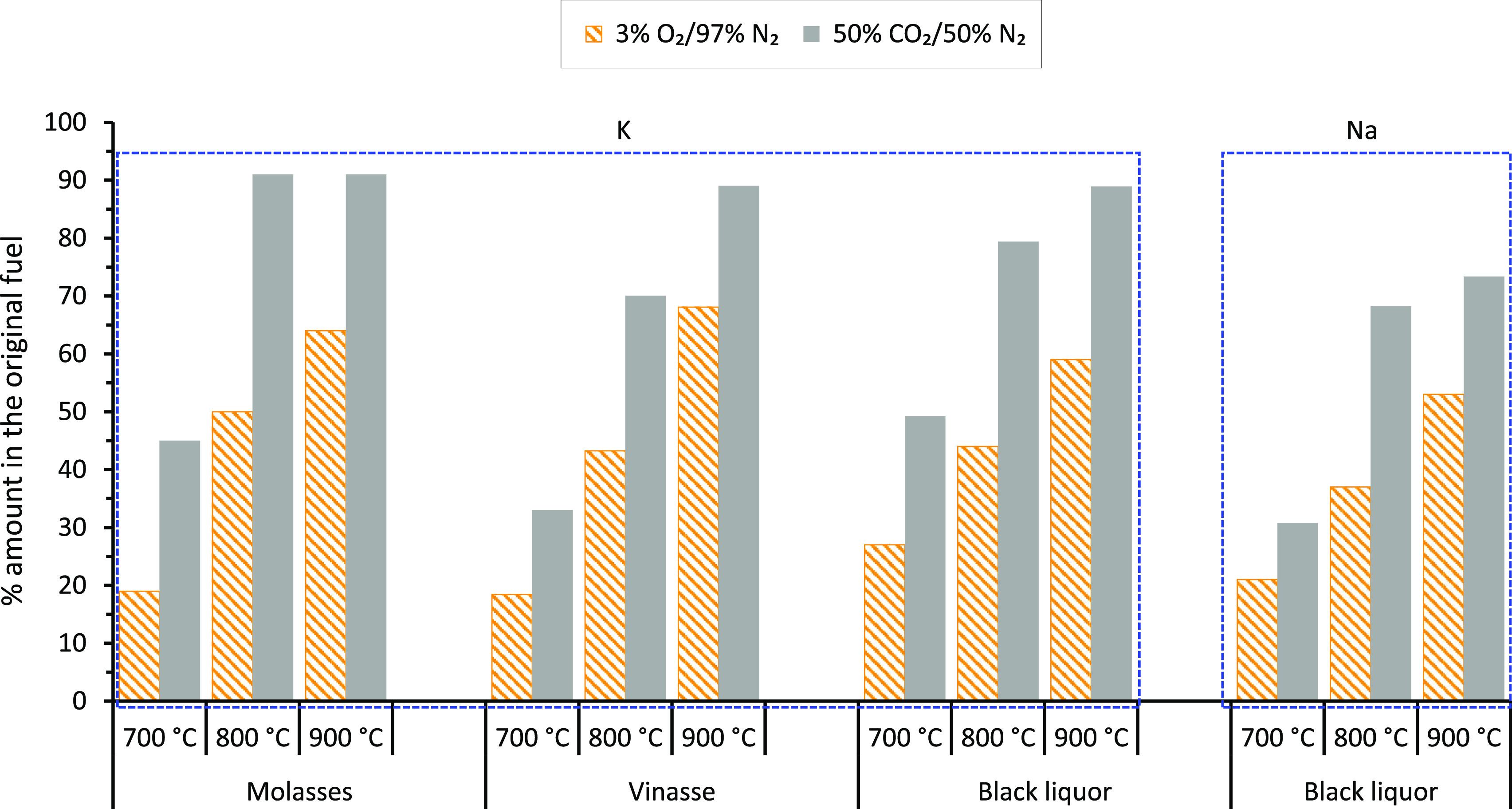
K and Na release results during combustion and gasification of molasses, vinasse, and black liquor at 700, 800, and 900 °C in the SPR.

**Figure 10:**
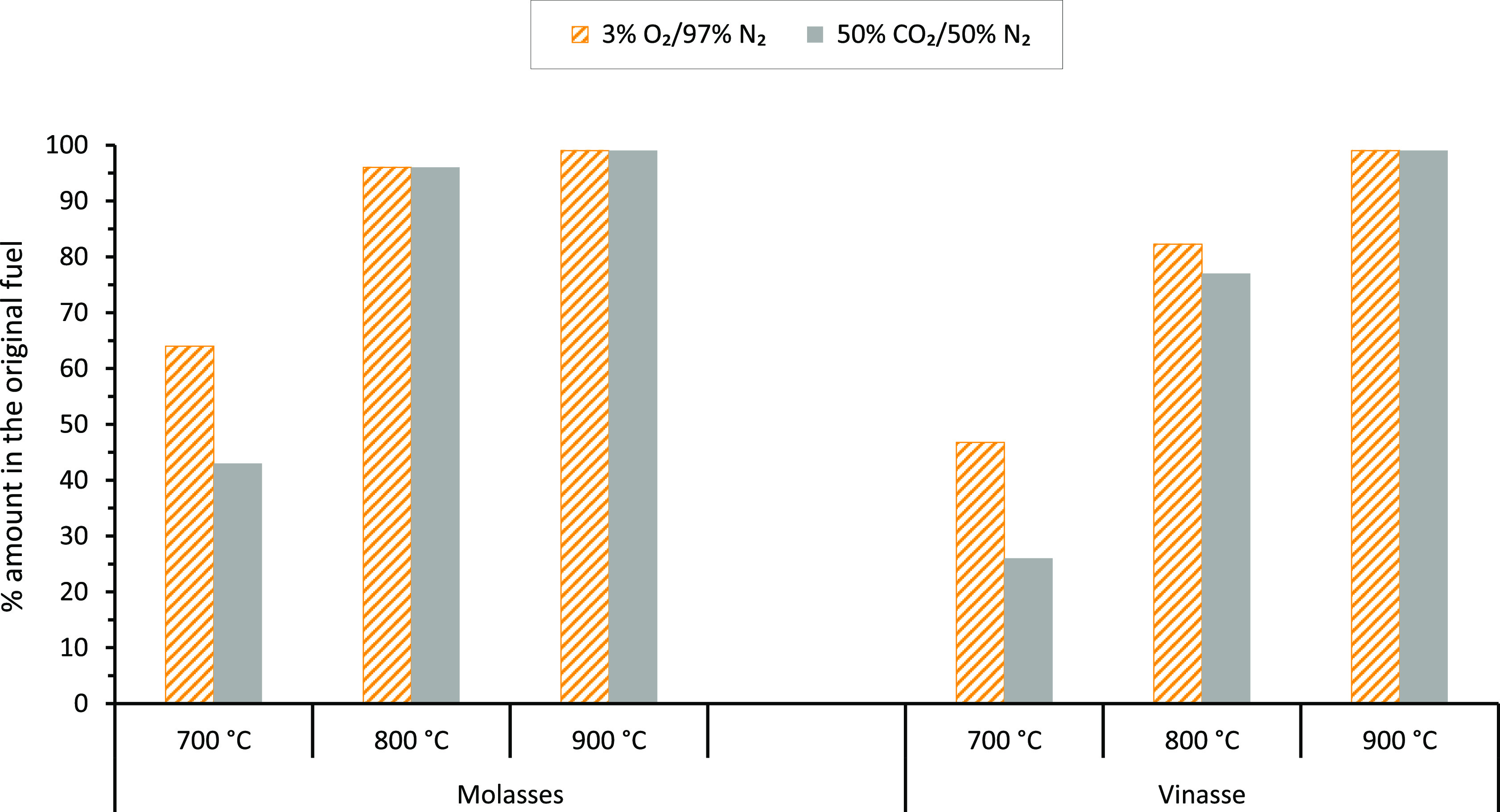
Cl release results during combustion and gasification of molasses and vinasse at 700, 800, and 900 °C in the SPR.

Figure 9 shows the K release results during combustion and gasification of the molasses, vinasse, and black liquor at 700, 800, and 900 °C in the SPR. The corresponding Cl release results are shown in Figure 10. The results shown in the figures were obtained from the difference between the Na, K, and Cl contents of the parent fuels listed in Table 2, Section 3.1, and the Na, K, and Cl contents of their corresponding ashes given in Table 4, Section 3.5. The Na release results for the molasses and vinasse and Cl for the black liquor were not reported here for the reason explained in the previous section, Section 3.5.

Figure 9 shows that about 20, 50, and 65% of the K in the molasses was released during combustion of the molasses in the SPR at 700, 800, and 900 °C, respectively. The corresponding K release numbers from the molasses under gasification conditions in the SPR increased to about 45 and 90% at 700 °C and ≥800 °C, respectively. The K release from the molasses trended with temperature similar to the K (and Na) release from the vinasse and black liquor samples under the same gas conditions, indicating similar alkali metal release mechanisms from the fuels. These mechanisms include evaporation of alkali metal salts^12,56^ and vaporization of the alkali metals after reduction on carbon.^57−59^ In addition, organically bound alkali metal ions can also be released from the fuels as vapors: MOH(g) under oxidizing conditions and M(g) under reducing environments,^60^ where M is Na and/or K. The rates at which these mechanisms occur increase with increasing temperature resulting in the increased release of the alkali metals at higher temperatures, as shown in the figure. The higher release numbers for the alkali metals from the fuels under gasification conditions than combustion are mainly due to two factors. First, the volatilization of the alkali metal vapors after reduction on carbon is more pronounced in the relatively reducing CO_2_ gas atmosphere than in the oxidizing O_2_ gas. A similar observation was reported previously for alkali release from vinasse.^19^ The mechanism of successive reduction of alkali carbonates on carbon and ultimately volatilization of the alkali metal vapors is well studied and available in the literature.^57−59^ Second, the relatively longer holding times used during gasification than combustion may have increased the alkali release. Several studies, for example, refs (12, 61), show that holding times strongly influence the release of alkali metals during biomass thermochemical conversion.

Figure 10 shows that the percentages of Cl released from the molasses at 700 °C in the SPR under combustion and gasification conditions were about 65 and 45% of the Cl in the molasses, respectively. At 800 and 900 °C, Cl was almost entirely released from the molasses, irrespective of the gas atmosphere used in the SPR. As shown in the figure, the Cl release trend with temperature for the molasses is similar to that of the vinasse. However, the percentages of Cl released at 700 and 800 °C from the molasses were higher than the corresponding amounts released from the vinasse under both gas conditions. This indicates that the fraction of volatile Cl in the molasses is higher than the proportion in the vinasse. In addition, Figure 10 shows that unlike with the alkali metals, more Cl was released in O_2_ than in CO_2_ gas from the molasses (at 700 °C) and vinasse (at 700 and 800 °C). A similar observation was reported for Cl release from vinasse in our previous study,^25^ where more Cl was released in a N_2_ gas atmosphere than in a relatively oxidizing CO_2_ gas atmosphere. In addition to Cl release as alkali chlorides by evaporation, described above for the release of alkali metals, Cl was probably also released as HCl(g) from the molasses and vinasse. For other biomass fuels,^62^ 20–60% of Cl in the fuels was released as HCl(g) during thermal conversion of the fuels below 500 °C.

The release of most of the K and Cl from the molasses under both combustion and gasification conditions suggests that molasses is a challenging fuel for conventional combustion and gasification processes. Nonetheless, the release of these elements from the molasses to a similar degree as from the vinasse shows that the thermal conversion options recommended for the vinasse may also be used for the molasses. These options and their pros and cons were described in detail for the vinasse in our previous study.^12^ Here, two of the main potential thermal conversion alternatives for the molasses are briefly described.

One option is combustion of the molasses in a black liquor recovery boiler type with strong oxidizing conditions in its lower furnace where the molasses can be fed into the boiler in coarse droplets. With this option, most of the ash-forming elements from the molasses can be contained in the bottom ash, thereby minimizing the risk of ash-related problems in superheater tubes, and at the same time, enabling the recovery of inorganic chemicals from the molasses with the bottom ash. Another alternative is gasification of the molasses in a very high temperature-entrained flow gasifier. This process involves feeding the molasses with oxygen into the gasifier, converting the organic fraction of the molasses into syngas in the reactor, and recovering most of the inorganics from the molasses as an aqueous-phase bottom product. The process has high carbon conversion efficiency, and it has been demonstrated to be a viable option, on a pilot-scale level, for syngas production and recovery of inorganic chemicals from black liquor.

### 3.7 Ash-Melting Behavior

Table 5 lists the results of characteristic ash-melting temperatures from the FactSage calculations and DSC measurements for the molasses, vinasse, and black liquor. Results of the ash melt fraction as a function of temperature obtained from the FactSage calculations are provided in Figure S2 of the Supporting Information. The calculated results given in Table 5 (and Figure S2) are based on the elemental compositions of the fuels given in Table 2, Section 3.1, and their ashes produced at 500 °C given in Table 1, Section 2.9.

**Table 5:** Results of Characteristic Ash-Melting Temperatures for the Molasses, Vinasse, and Black Liquor (°C)

ash	calculated				measured
	*T*_0_	*T*_15_	*T*_70_	*T*_100_	*T*_0_
molasses	640–650	650–665	670–720		640
vinasse^a^	640-645	650	670-690		640
black liquor	570–575	650–700	755–760	775	565

a Data are from ref (19).

As seen from Table 5, the *T*_0_ results obtained from the FactSage calculations agree with the values obtained from the DSC measurements for all the fuels. The results given in the table (and in Figure S2) can be summarized as follows. (a) The molasses and vinasse ashes had similar characteristic ash-melting temperatures. The similar ash-melting temperatures for the molasses and vinasse ashes arise from the fact that molasses is a feedstock for bioethanol production and vinasse is the final byproduct obtained from the distillery. As a result, in the process of fermenting the molasses and distilling ethanol from the fermented mass, most of the ash-forming elements in the molasses end up in the vinasse. For example, we reported in our previous study^2^ that about 90% of K and 80% of Cl from the molasses culminate in the vinasse. (b) Similar to the vinasse ash, the molasses ash did not melt 100% below 1000 °C, that is, it was not possible to determine *T*_100_ for the ash. This was due to the small amounts of high-temperature melting components in the ash, mainly Ca. However, the black liquor ash was 100% molten at about 775 °C. (c) 70% of the molasses ash was melted in a narrow temperature range, 640–720 °C, roughly similar to that of the vinasse ash, indicating that the molasses ash is dominantly composed of an ash-forming compound, probably KCl. In the vinasse ash, KCl was the main component.^19^ However, the black liquor ash melted in a broader temperature range. (d) The FactSage thermodynamic calculation results (Figure S2) show that the melt fraction for the molasses ash starts to decrease with temperature after 750 °C, likely due to evaporation of the alkali metal salts as described in Section 3.6.

Here too, the low ash-melting properties of the molasses ash suggest that molasses is a problematic fuel to utilize using existing thermal conversion technologies. Nevertheless, the similar ash-melting characteristics of the molasses ash with those of the vinasse ash indicate that the thermal conversion options recommended for the vinasse and described in Section 3.6 may also be applied for the molasses.

## 4 Conclusions

In this study, thermal conversion characteristics of molasses have been studied using a lab-scale SPR at temperatures of 700–900 °C. Swelling during pyrolysis, combustion times, CO gas yields and char gasification reactivities, and NO emissions and release of K and Cl under combustion and gasification conditions were the thermal conversion properties of the molasses studied. In addition, the melting behavior of molasses ash produced at 500 °C was assessed using FactSage thermodynamic modeling and DSC measurements. Results of the molasses thermal conversion characteristics were compared with those of vinasse and black liquor after conducting experiments using samples of the fuels under similar experimental conditions as those of the molasses. The following conclusions can be drawn from the present study.

(a) Compared to the vinasse and black liquor, the molasses had the least swelling tendency and the longest combustion duration in the SPR, suggesting its slower conversion in an industrial boiler than the vinasse and black liquor. Moreover, the gasification rates of the molasses at ≥800 °C were lower than those of the vinasse and black liquor, probably owing to the lower total concentration of catalytic alkali and alkaline earth metals in the molasses. However, higher CO gas yields were obtained from the molasses in the temperature range used in this study.
(b) The NO emission from the molasses trended with temperature in a fashion similar to the NO emissions from the vinasse and black liquor. The fuel-N release as NO increased, almost linearly, with temperature under combustion conditions, while the release remained approximately constant, that is, below 5–10% of the fuel-N, with temperatures in a CO_2_ gas atmosphere.
(c) Results of analysis of the ashes from the combustion and gasification experiments in the SPR showed that significant levels of K and Cl were released from the molasses, and the release levels increased considerably with the temperature. The alkali metal(s) release was higher in the CO_2_ gas atmosphere than in O_2_ for all the fuels, but a higher Cl release was observed in O_2_ than in CO_2_. Cl was effectively released from the molasses at ≥800 °C, irrespective of the gas atmosphere.
(d) The FactSage thermodynamic modeling and DSC measurements revealed that the molasses ash had a very similar melting behavior as that of the vinasse ash.
(e) The release of K and Cl to a high degree from the molasses during combustion and gasification and the low melting temperature of the molasses ash make the molasses a challenging fuel to utilize using the existing thermal conversion technologies. Nevertheless, a black liquor recovery boiler type with a simpler (or an oxidizing) lower furnace compared to a black liquor recovery boiler and an entrained flow gasifier of the type demonstrated for black liquor may be potential options for the production of energy and recovery of inorganic chemicals from the molasses.

## Supporting Information Available

Results of fuel-N release as NO from the molasses, vinasse, and black liquor as a function of fuel-N content (wt % dry fuel) (Figure S1) and melt fraction as a function of temperature from the FactSage calculations for the molasses, vinasse, and black liquor and their ashes produced at 500 °C (Figure S2) (PDF)

## References

[ref1] *Directive (EU) 2018/2001*; http://data.europa.eu/eli/dir/2018/2001/2018-12-21 (accessed on May 27, 2021).

[ref2] Dirbeba M. J.; Brink A.; DeMartini N.; Zevenhoven M.; Hupa M. Potential for Thermochemical Conversion of Biomass Residues from the Integrated Sugar-Ethanol Process — Fate of Ash and Ash-Forming Elements. Bioresour. Technol. 2017, 234, 188–197. 10.1016/j.biortech.2017.03.021. 28319767

[ref3] Rein P. Cane Sugar Engineering, 1st ed.; Verlag Dr. Albert Bartens KG: Berlin, 2007.

[ref4] http://www.fao.org/faostat/ (accessed on May 26, 2021).

[ref5] Fuess L. T.; Zaiat M.; do Nascimento C. A. O. Molasses vs. Juice: Maximizing Biohydrogen Production in Sugarcane Biorefineries to Diversify Renewable Energy Generation. J. Water Process Eng. 2020, 37, 101534 10.1016/j.jwpe.2020.101534.

[ref6] Fuess L. T.; Garcia M. L. Anaerobic Digestion of Stillage to Produce Bioenergy in the Sugarcane-to-Ethanol Industry. Environ. Technol. 2014, 35, 333–339. 10.1080/09593330.2013.827745. 24600872

[ref7] Pimentel D.; Patzek T. W. Ethanol Production: Energy and Economic Issues Related to U.S. and Brazilian Sugarcane. In Biofuels, Solar and Wind as Renewable Energy Systems: Benefits and Risks; Pimentel D., Ed.; Springer: Dordrecht, 2008; 357–371. 10.1007/978-1-4020-8654-0_14.

[ref8] Atlason R.; Unnthorsson R. Ideal EROI (Energy Return on Investment) Deepens the Understanding of Energy Systems. Energy 2014, 67, 241–245. 10.1016/j.energy.2014.01.096.

[ref9] de Oliveira B. G.; Carvalho J. L. N.; Cerri C. E. P.; Cerri C. C.; Feigl B. J. Soil Greenhouse Gas Fluxes from Vinasse Application in Brazilian Sugarcane Areas. Geoderma 2013, 200-201, 77–84. 10.1016/j.geoderma.2013.02.005.

[ref10] Christofoletti C. A.; Escher J. P.; Correia J. E.; Marinho J. F. U.; Fontanetti C. S. Sugarcane Vinasse: Environmental Implications of Its Use. Waste Manage. 2013, 33, 2752–2761. 10.1016/j.wasman.2013.09.005. 24084103

[ref11] Fuess L. T.; Fuentes L.; Bovio-Winkler P.; Eng F.; Etchebehere C.; Zaiat M.; Do Nascimento C. A. O. Full Details on Continuous Biohydrogen Production from Sugarcane Molasses Are Unraveled: Performance Optimization, Self-Regulation, Metabolic Correlations and Quanti-Qualitative Biomass Characterization. Chem. Eng. J. 2021, 414, 128934 10.1016/j.cej.2021.128934.

[ref12] Dirbeba M. J. Thermochemical Conversion Characteristics of Vinasse (Ph.D. Thesis), Åbo Akademi, 2020.

[ref13] Adams T. N.; Frederick W. J.; Grace T. M.; Hupa M.; Iisa K.; Jones A. K.; Tran H. Kraft Recovery Boilers; Adams T. N., Ed.; Tappi Press: Atlanta, 1997.

[ref14] Frederick W. J.; Noopila T.; Hupa M. Swelling of Pulping Liquor Droplets during Combustion. J. Pulp Pap. Sci. 1991, 17, J164–J170.

[ref15] Whitty K. Pyrolysis and Gasification Behavior of Black Liquor under Pressurized Conditions (Ph.D. Thesis), Åbo Akademi University, 1997.

[ref16] Baxter L. L.; Miles T. R.; Miles T. R. Jr.; Jenkins B. M.; Milne T.; Dayton D.; Bryers R. W.; Oden L. L. The Behavior of Inorganic Material in Biomass-Fired Power Boilers: Field and Laboratory Experiences. Fuel Process. Technol. 1998, 54, 47–78. 10.1016/S0378-3820(97)00060-X.

[ref17] Backman R.; Skrifvars B.-J.; Hupa M.; Siiskonen P.; Mäntyniemi J. Flue Gas and Dust Chemistry in Recovery Boilers with High Levels of Chlorine and Potassium. J. Pulp Pap. Sci. 1996, 22, J119–J126.

[ref18] Tran H. N.; Reeve D. W.; Barham D. Formation of Kraft Recovery Boiler Superheater Fireside Deposits. Pulp Pap. Canada 1983, 84, T7–T12.

[ref19] Dirbeba M. J.; Brink A.; Zevenhoven M.; Demartini N.; Lindberg D.; Hupa L.; Hupa M. Characterization of Vinasse for Thermochemical Conversion — Fuel Fractionation, Release of Inorganics, and Ash-Melting Behavior. Energy Fuels 2019, 33, 5840–5848. 10.1021/acs.energyfuels.8b04177.

[ref20] Hupa M.; Karlström O.; Vainio E. Biomass Combustion Technology Development - It Is All about Chemical Details. Proc. Combust. Inst. 2017, 36, 113–134. 10.1016/j.proci.2016.06.152.

[ref21] Leckner B.; Karlsson M.; Dam-Johansen K.; Weinell C. E.; Kilpinen P.; Hupa M. Influence of Additives on Selective Noncatalytic Reduction of NO with NH_3_ in Circulating Fluidized Bed Boilers. Ind. Eng. Chem. Res. 1991, 30, 2396–2404. 10.1021/ie00059a006.

[ref22] Arand J. K.; Muzio L. J.; Sotter J. G. Urea Reduction of NOx in Combustion Effluents. U.S. Patent 4,208,386 A, June 17, 1980.

[ref23] Dirbeba M. J.; Aho A.; Demartini N.; Brink A.; Hupa M. Pyrolysis of Sugarcane Vinasse and Black Liquor at 400 and 500 °C. In Proc. 13th Int. Conf. Energ. Clean Env.; São Miguel, Azores, Portugal, July 2–6, 2017.

[ref24] Torquato L. D. M.; Crnkovic P. M.; Ribeiro C. A.; Crespi M. S. New Approach for Proximate Analysis by Thermogravimetry Using CO_2_ Atmosphere: Validation and Application to Different Biomasses. J. Therm. Anal. Calorim. 2017, 128, 1–14. 10.1007/s10973-016-5882-z.

[ref25] Dirbeba M. J.; Brink A.; DeMartini N.; Lindberg D.; Hupa M. Sugarcane Vinasse CO_2_ Gasification and Release of Ash-Forming Matters in CO_2_ and N_2_ Atmospheres. Bioresour. Technol. 2016, 218, 606–614. 10.1016/j.biortech.2016.07.004. 27403861

[ref26] Dirbeba M. J.; Aho A.; Demartini N.; Brink A.; Mattsson I.; Hupa L.; Hupa M. Fast Pyrolysis of Dried Sugar Cane Vinasse at 400 and 500 °C: Product Distribution and Yield. Energy Fuels 2019, 33, 1236–1247. 10.1021/acs.energyfuels.8b03757.

[ref27] Giuntoli J.; de Jong W.; Verkooijen A. H. M.; Piotrowska P.; Zevenhoven M.; Hupa M. Combustion Characteristics of Biomass Residues and Biowastes: Fate of Fuel Nitrogen. Energy Fuels 2010, 24, 5309–5319. 10.1021/ef100571n.

[ref28] Karlström O.; Brink A.; Hupa M. Time Dependent Production of NO from Combustion of Large Biomass Char Particles. Fuel 2013, 103, 524–532. 10.1016/j.fuel.2012.06.030.

[ref29] Perander M.; DeMartini N.; Brink A.; Kramb J.; Karlström O.; Hemming J.; Moilanen A.; Konttinen J.; Hupa M. Catalytic Effect of Ca and K on CO_2_ Gasification of Spruce Wood Char. Fuel 2015, 150, 464–472. 10.1016/j.fuel.2015.02.062.

[ref30] Bale C. W.; Bélisle E.; Chartrand P.; Decterov S. A.; Eriksson G.; Hack K.; Jung I.-H.; Kang Y.-B.; Melançon J.; Pelton A. D.; Robelin C.; Petersen S. FactSage Thermochemical Software and Databases — Recent Developments. Calphad 2009, 33, 295–311. 10.1016/j.calphad.2008.09.009.

[ref31] Lindberg D.; Chartrand P. Thermodynamic Evaluation and Optimization of the (Ca + C + O + S) System. J. Chem. Thermodyn. 2009, 41, 1111–1124. 10.1016/j.jct.2009.04.018.

[ref32] Chen Y.; Mori S.; Pan W. P. Studying the Mechanisms of Ignition of Coal Particles by TG-DTA. Thermochim. Acta 1996, 275, 149–158. 10.1016/0040-6031(95)02727-0.

[ref33] Grotkjær T.; Dam-Johansen K.; Jensen A. D.; Glarborg P. An Experimental Study of Biomass Ignition. Fuel 2003, 82, 825–833. 10.1016/S0016-2361(02)00369-1.

[ref34] Basu P. Biomass Gasification and Pyrolysis, 1st ed.; Academic Press: Burlington, MA, 2010. 10.1016/C2009-0-20099-7.

[ref35] Hupa M.; Solin P.; Hyöty P. Combustion of Black Liquor Droplets. J. Pulp Pap. Sci. 1987, 13, J67–J72.

[ref36] Soderhjelm L.; Hupa M.; Noopila T. Combustibility of Black Liquors with Different Rheological and Chemical Properties. J. Pulp Pap. Sci. 1989, 15, J117–J121.

[ref37] Miller P. T.; Clay D. T.; Lonsky W. F. W. The Influence of Composition on the Swelling of Kraft Black Liquor during Pyrolysis. Chem. Eng. Commun. 1989, 101–120. 10.1080/00986448908940671.

[ref38] Ahmed I.; Gupta A. K. Characteristics of Cardboard and Paper Gasification with CO_2_. Appl. Energy 2009, 86, 2626–2634. 10.1016/j.apenergy.2009.04.002.

[ref39] Hanaoka T.; Inoue S.; Uno S.; Ogi T.; Minowa T. Effect of Woody Biomass Components on Air-Steam Gasification. Biomass Bioenergy 2005, 28, 69–76. 10.1016/j.biombioe.2004.03.008.

[ref40] Nam H.; Wang S.; Sanjeev K. C.; Seo M. W.; Adhikari S.; Shakya R.; Lee D.; Shanmugam S. R. Enriched Hydrogen Production over Air and Air-Steam Fluidized Bed Gasification in a Bubbling Fluidized Bed Reactor with CaO: Effects of Biomass and Bed Material Catalyst. Energy Convers. Manage. 2020, 225, 113408 10.1016/j.enconman.2020.113408.

[ref41] Billaud J.; Valin S.; Peyrot M.; Salvador S. Influence of H_2_O, CO_2_ and O_2_ Addition on Biomass Gasification in Entrained Flow Reactor Conditions: Experiments and Modelling. Fuel 2016, 166, 166–178. 10.1016/j.fuel.2015.10.046.

[ref42] Sadhwani N.; Adhikari S.; Eden M. R. Biomass Gasification Using Carbon Dioxide: Effect of Temperature, CO_2_/C Ratio, and the Study of Reactions Influencing the Process. Ind. Eng. Chem. Res. 2016, 55, 2883–2891. 10.1021/acs.iecr.5b04000.

[ref43] Li P.; Miser D. E.; Rabiei S.; Yadav R. T.; Hajaligol M. R. The Removal of Carbon Monoxide by Iron Oxide Nanoparticles. Appl. Catal. B Environ. 2003, 43, 151–162. 10.1016/S0926-3373(02)00297-7.

[ref44] Lahijani P.; Zainal Z. A.; Mohamed A. R.; Mohammadi M. CO_2_ Gasification Reactivity of Biomass Char: Catalytic Influence of Alkali, Alkaline Earth and Transition Metal Salts. Bioresour. Technol. 2013, 144, 288–295. 10.1016/j.biortech.2013.06.059. 23880130

[ref45] Wang G.; Zhang J.; Shao J.; Liu Z.; Wang H.; Li X.; Zhang P.; Geng W.; Zhang G. Experimental and Modeling Studies on CO_2_ Gasification of Biomass Chars. Energy 2016, 114, 143–154. 10.1016/j.energy.2016.08.002.

[ref46] Cetin E.; Gupta R.; Moghtaderi B. Effect of Pyrolysis Pressure and Heating Rate on Radiata Pine Char Structure and Apparent Gasification Reactivity. Fuel 2005, 84, 1328–1334. 10.1016/j.fuel.2004.07.016.

[ref47] Fushimi C.; Araki K.; Yamaguchi Y.; Tsutsumi A. Effect of Heating Rate on Steam Gasification of Biomass. 1. Reactivity of Char. Ind. Eng. Chem. Res. 2003, 42, 3922–3928. 10.1021/ie030056c.

[ref48] Karlström O.; Dirbeba M. J.; Costa M.; Brink A.; Hupa M. Influence of K/C Ratio on Gasification Rate of Biomass Chars. Energy Fuels 2018, 32, 10695–10700. 10.1021/acs.energyfuels.8b02288.

[ref49] Bhatia S. K.; Perlmutter D. D. A Random Pore Model for Fluid-solid Reactions: I. Isothermal, Kinetic Control. AIChE J. 1980, 379–386. 10.1002/aic.690260308.

[ref50] Vähä-Savo N.; Demartini N.; Engblom M.; Brink A.; Hupa M. The Fate of Char Nitrogen in Black Liquor Combustion—Cyanate Formation and Decomposition. Ind. Eng. Chem. Res. 2015, 54, 2831–2842. 10.1021/ie503450r.

[ref51] Vainio E. Fate of Fuel-Bound Nitrogen and Sulfur in Biomass-Fired Industrial Boilers (Ph.D. Thesis), Åbo Akademi University, 2014.

[ref52] Vähä-Savo N. Behavior of Black Liquor Nitrogen in Combustion - Formation of Cyanate (Ph.D. Thesis), Åbo Akademi University, Turku, Finland, 2014.

[ref53] Aho K. Nitrogen Oxides Formation in Recovery Boilers (Licentiate Thesis), Åbo Akademi University, 1994.

[ref54] Salzmann R.; Nussbaumer T. Fuel Staging for NOx Reduction in Biomass Combustion: Experiments and Modelling. Energy Fuels 2001, 15, 575–582. 10.1021/ef0001383.

[ref55] Watanabe H.; Shimomura K.; Okazaki K. Carbonate Formation during Lignin Pyrolysis under CO_2_ and Its Effect on Char Oxidation. Proc. Combust. Inst. 2015, 35, 2423–2430. 10.1016/j.proci.2014.06.014.

[ref56] Knudsen J. N.; Jensen P. A.; Dam-Johansen K. Transformation and Release to the Gas Phase of Cl, K, and S during Combustion of Annual Biomass. Energy Fuels 2004, 18, 1385–1399. 10.1021/ef049944q.

[ref57] Sams D. A.; Shadman F. Mechanism of Potassium-Catalyzed Carbon/CO_2_ Reaction. AIChE J. 1986, 32, 1132–1137. 10.1002/aic.690320710.

[ref58] Kopyscinski J.; Rahman M.; Gupta R.; Mims C. A.; Hill J. M. K_2_CO_3_ Catalyzed CO_2_ Gasification of Ash-Free Coal. Interactions of the Catalyst with Carbon in N_2_ and CO_2_ Atmosphere. Fuel 2014, 117, 1181–1189. 10.1016/j.fuel.2013.07.030.

[ref59] Kapteijn F.; Moulijn J. A. Kinetics of the Potassium Carbonate-Catalysed CO_2_ Gasification of Activated Carbon. Fuel 1983, 62, 221–225. 10.1016/0016-2361(83)90203-X.

[ref60] Zevenhoven M.; Yrjas P.; Hupa M. Ash-Forming Matter and Ash-Related Problems. In Handbook of combustion: Part 4. Solid fuels; Lackner M., Winter F., Agarwal A. K., Eds.; WILEY-VCH Verlag GmbH & Co. KGaA: Weinheim, 2010; 493–531.

[ref61] Okuno T.; Sonoyama N.; Hayashi J.; Li C.-Z.; Sathe C.; Chiba T. Primary Release of Alkali and Alkaline Earth Metallic Species during the Pyrolysis of Pulverized Biomass. Energy Fuels 2005, 19, 2164–2171. 10.1021/ef050002a.

[ref62] Jensen P. A.; Frandsen F. J.; Dam-Johansen K.; Sander B. Experimental Investigation of the Transformation and Release to Gas Phase of Potassium and Chlorine during Straw Pyrolysis. Energy Fuels 2000, 14, 1280–1285. 10.1021/ef000104v.

