# Supplementary material for: Thermal Conversion Characteristics of Molasses
Source: ACS Omega. 2021 Aug 10;6(33):21631–45. doi: 10.1021/acsomega.1c03024 (PMC8388098; doi:10.1021/acsomega.1c03024)
Supplement: Supplementary file 1 — ao1c03024_si_001.pdf [file ao1c03024_si_001.pdf]

Supporting Information

# Thermal Conversion Characteristics of Molasses

*Meheretu Jaleta Dirbeba*[†,*], *Anders Brink*[†], *Daniel Lindberg*[‡], *Mikko Hupa*[†], *Leena Hupa*[†]

[†]Johan Gadolin Process Chemistry Centre, Åbo Akademi University, Henrikinkatu 2, 20500 Turku/Åbo, Finland

[‡]Department of Chemical and Metallurgical Engineering, Aalto University, Kemistintie 1, P.O.Box 11000, 00076 Aalto, Espoo, Finland

[*]Corresponding Author: E-mail: meheretu.dirbeba@abo.fi

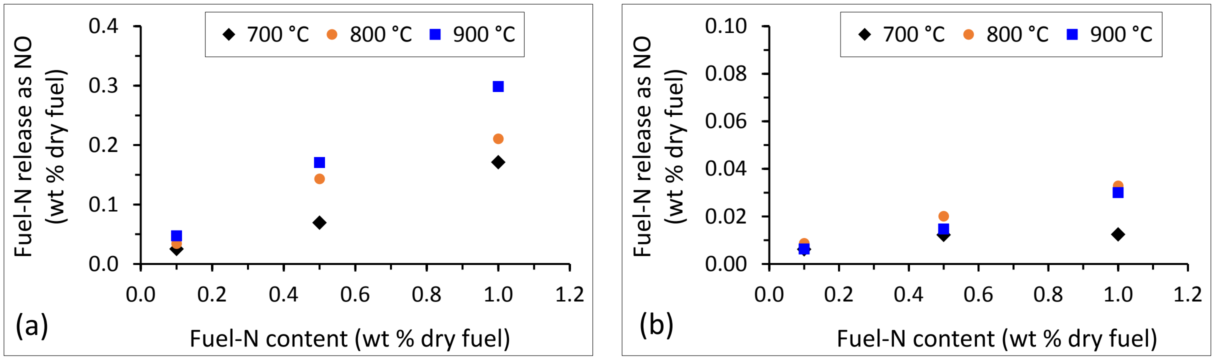


**Figure S1.** Results of fuel-N release as NO from the molasses, vinasse, and black liquor as a function of fuel-N content (wt % dry fuel).

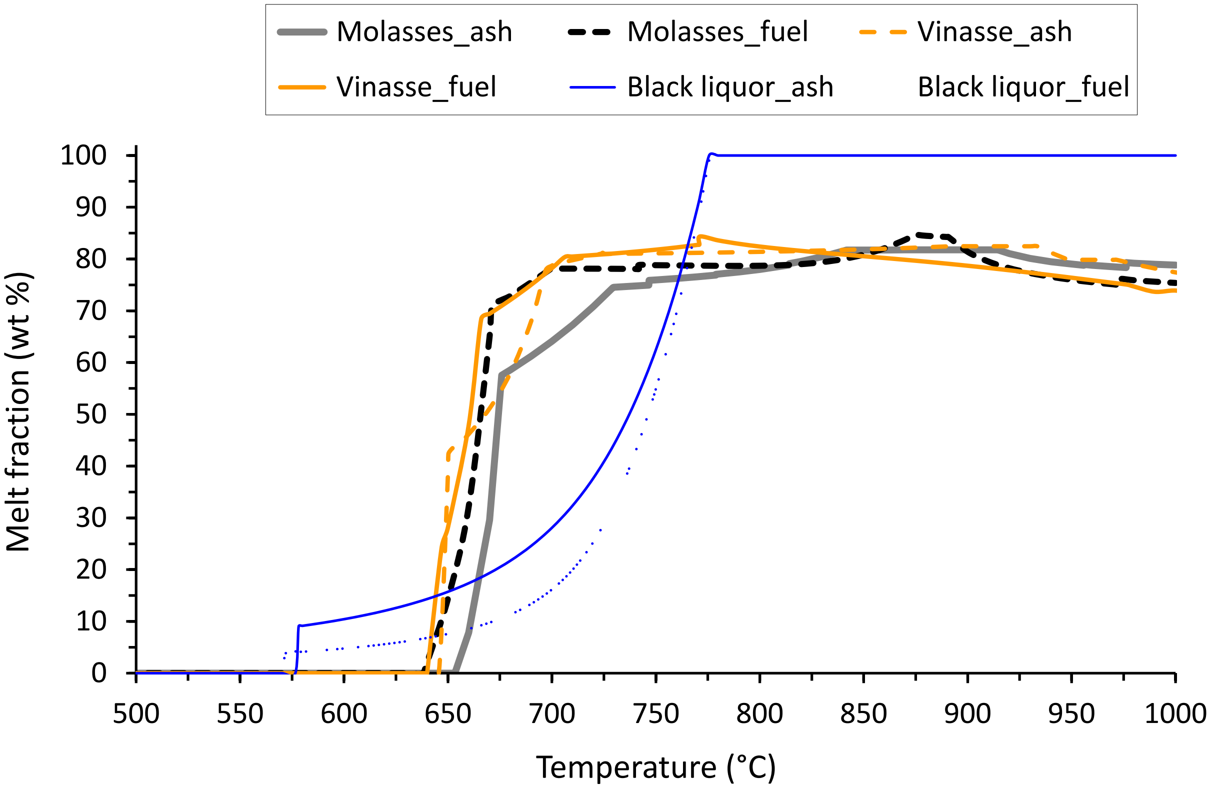


**Figure S2.** Melt fraction as a function of temperature from the FactSage calculations for the molasses, vinasse, and black liquor and their ashes produced at 500 °C
